# Impact of molecular tumour board discussion on targeted therapy allocation in advanced prostate cancer

**DOI:** 10.1038/s41416-021-01663-9

**Published:** 2021-12-15

**Authors:** Peter H. J. Slootbeek, Iris S. H. Kloots, Minke Smits, Inge M. van Oort, Winald R. Gerritsen, Jack A. Schalken, Marjolijn J. L. Ligtenberg, Katrien Grünberg, Leonie I. Kroeze, Haiko J. Bloemendal, Niven Mehra

**Affiliations:** 1 Radboud University Medical Centre, Radboud Institute for Health Sciences, Department of Medical Oncology, Nijmegen, The Netherlands; 2 Radboud University Medical Centre, Radboud institute for Molecular Life sciences, Department of Experimental Urology, Nijmegen, The Netherlands; 3 Radboud University Medical Centre, Radboud Institute for Health Sciences, Department of Urology, Nijmegen, The Netherlands; 4 Radboud University Medical Centre, Radboud Institute for Molecular Life sciences, Department of Pathology, Nijmegen, The Netherlands; 5 Radboud University Medical Centre, Radboud Institute for Molecular Life sciences, Department of Human Genetics, Nijmegen, The Netherlands

**Keywords:** Prostate cancer, Cancer genetics, Targeted therapies, Cancer immunotherapy

## Abstract

**Background:**

Molecular tumour boards (MTB) optimally match oncological therapies to patients with genetic aberrations. Prostate cancer (PCa) is underrepresented in these MTB discussions. This study describes the impact of routine genetic profiling and MTB referral on the outcome of PCa patients in a tertiary referral centre.

**Methods:**

All PCa patients that received next-generation sequencing results and/or were discussed at an MTB between Jan 1, 2017 and Jan 1, 2020 were included. Genetically matched therapies (GMT) in clinical trials or compassionate use were linked to actionable alterations. Response to these agents was retrospectively evaluated.

**Results:**

Out of the 277 genetically profiled PCa patients, 215 (78%) were discussed in at least one MTB meeting. A GMT was recommended to 102 patients (47%), of which 63 patients (62%) initiated the GMT. The most recommended therapies were PARP inhibitors (*n* = 74), programmed death-(ligand) 1 inhibitors (*n* = 21) and tyrosine kinase inhibitors (*n* = 19). Once started, 41.3% had a PFS of ≥6 months, 43.5% a PSA decline ≥50% and 38.5% an objective radiographic response.

**Conclusion:**

Recommendation for a GMT is achieved in almost half of the patients with advanced prostate cancer, with GMT initiation leading to durable responses in over 40% of patients. These data justify routine referral of selected PCa patients to MTB’s.

## Introduction

Molecular tumour boards (MTB) aid physicians in deciphering an increasingly complex and evolving druggable genomic landscape of cancer. All with the goal of identifying genetic aberrations associated with susceptibility or resistance to targeted therapies or immunotherapy [1]. Their role in oncological care has grown in parallel with the rise of precision oncology. Although several recommendations are proposed to standardise and optimise the running of an MTB [2–5], the structure still varies among institutes [6–17]. In all studies, one of the main aims of the MTB is to provide a recommendation for a genetically matched therapy (GMT) [18]. Although commonly more than half of the discussed patients harboured a druggable genetic aberration, only a minority initiated their recommended GMT [18, 19]. This may be explained by a high proportion of patients not eligible for trial inclusion due to a decline in performance status and organ function as a result of discussion too late in the course of their disease state [20]. The first, the third and the fourth most diagnosed solid cancers (breast, lung & bronchus and colon & rectum) account for the majority of MTB discussions [21]. Intriguingly, prostate cancer (PCa), the second-most diagnosed cancer, is rarely discussed. For instance, in the prospective analysis of the UK Genomics Review Board, only four of the 895 patients that were discussed were PCa cases [11]. Dalton et al. described 155 patients that were discussed within the Johns Hopkins tumour board, of which two with PCa [8] and in the multicentre ProfiLER trial with 2579 patients, 53 were genito-urinary cancer cases [17]. To our knowledge, the Curie institute reported the highest number of PCa patient discussions within an MTB, namely 37 patients (5%) [9].

This is remarkable since in 2015 it was shown by Robinson et al. that nearly 90% of all metastatic castration-resistant prostate cancer (mCRPC) patients harboured potentially actionable genetic events [22]. Excluding structural variants and mutations in *AR*, not yet considered actionable, still 65% of patients remain potentially targetable by a GMT. Moreover, the need for targeted therapies in mCRPC is high, since treatment options are limited and mCRPC patients inevitably develop resistance against the available standard of care. This contributes to prostate cancer being the second leading cancer-related cause of death in men, with only a minority of patients surviving longer than 3 years following the development of CRPC [21, 23]. Due to the notorious heterogeneity of mCRPC, a range of potential genetic targets can be exploited by GMTs. PARP inhibitors (PARPi) [24–27], platinum-based chemotherapy [28, 29], programmed death(-ligand) 1 inhibitors (PD-[L]1i) [30, 31] and more recently phosphoinositide 3-kinase/AKT (PI3K/AKT) blockade [32], all show significant antitumour activity in biomarker enriched mCRPC subgroups. In addition, basket trials, like the drug rediscovery protocol (DRUP) trial [33], the targeted agent and profiling utilisation registry (TAPUR) trial [34] and the ProTarget study facilitate the off-label use of commercially available targeted anticancer drugs for patients with a potentially actionable genomic aberration (NCT02925234, NCT02693535, NCT04341181).

Despite the availability of a broad armamentarium of GMTs and a clinical necessity to deliver these therapies to the right PCa patients, literature describing the impact of precision medicine through MTBs is lacking. The Radboudumc is a tertiary referral centre for prostate cancer and hosts an MTB for solid tumours since 2015. Most of the patients discussed are PCa patients. This retrospective single-centre study describes the results and clinical impact of routine genetic profiling and discussion of PCa patients at our MTB between 2017 and 2020.

## Methods

### MTB design

The Radboudumc MTB has weekly meetings, attended by oncologists, pathologists, clinical scientists in molecular pathology, clinical geneticists and pharmacists. Discussion reports, including the specified gene and GMT advice, were stored in the electronic health records of the patients. Recommendations were given based on recruiting trials within the Netherlands (e.g. DRUP trial, NCT02925234) and insights at the time of discussion based on the ESCAT framework [35]. Therefore, during the 3-year period, the basis for advice was subject to ever-evolving developments in the field. For instance, in our MTB, *PTEN* was in this time-frame not considered a druggable gene in prostate cancer, as trials with GMTs targeting AKT/PI3K were not running in the Netherlands, were not available off-label, and appeared to have relevance only when combined with androgen-receptor targeting agents. Off-label use of GMT, through compassionate use programmes, was only initiated if patients could not be included in clinical trials. GMT recommendation by the MTB consisted of either the advice to initiate GMT or the advice to further explore the possibility to obtain access to a specific GMT. Recommendations were given for germline testing based on the presence of somatic variants in cancer predisposition genes, also taking into account the patient’s family history. These recommendations did not impact GMT allocation, and therefore were not analysed in this study. Patients were automatically referred for MTB discussion following whole-genome sequencing or manually referred by their treating oncologist following the acquisition of any other form of molecular tests. A performance status or organ function inadequate to participate in clinical trials were reasons to avert from MTB referral.

### Genetic testing

In the MTB, next-generation sequencing (NGS) results from within the Radboudumc and by external laboratories were discussed. Archived or fresh tumour material was sequenced by the Pathology department (*n* = 146), using targeted NGS panels with either single-molecule molecular inversion probes or a capture-based approach incorporating unique molecular identifiers [36–38]. Externally, samples were whole-genome sequenced by the Hartwig Medical Foundation for the Centre for Personalised Cancer Treatment trial (CPCT, *n* = 152), and targeted sequenced by Foundation Medicine (FMI, *n* = 193). Gene panels are presented in Supplementary Appendix 2. CPCT reported only select genes by in silico filtering and filtered out germline variants depending on the consent choice of the patient. The number of reported genes increased over the inclusion period. FMI sequencing was performed within the scopes of the PROfound trial (NCT02987543) and TALAPRO-1 study (NCT03148795). To mitigate differences in pathogenicity reporting, all externally obtained sequencing reports were re-assessed largely based on guidelines from the American College of Medical Genetics and Genomics and the Association for Molecular Pathology [39, 40]. Patients who underwent biopsy for sequencing outside of routine care provided informed consent.

### Patient population

All included patients were treated at the Radboudumc and received successful NGS results between Jan 1, 2017 and Jan 1, 2020 or were discussed at the MTB during the same period. Patient follow-up was until Jan 1, 2021. Medical records were extracted and pseudo-anonymised demographic, clinical and molecular data were stored in an electronic database (www.castoredc.com), including the advice provided by the MTB.

### Statistical analysis and outcomes

Descriptive statistics were used to characterise the study population. Subgroups were compared using Fisher’s Exact test for categorical variables and the nonparametric Mann–Whitney *U* test for continuous variables.

Time-to-event data were estimated using the Kaplan–Meier method. Progression-free survival (PFS) was defined as the time from starting a GMT until the first moment of radiographic progression, clinical progression or death. Censoring took place at end of follow-up or at the initiation of the next systemic therapy. Biochemical PFS was defined as the time from initiation of GMT until a confirmed increase of >25% from the lowest prostate-specific antigen (PSA) value, ignoring early rises before 12 weeks where possible. Overall survival (OS) was defined as any cause of death, with censoring for non-deceased patients at the last follow-up date. To evaluate the effect of GMT utilisation on OS, only patients that had died and/or initiated at least one line of GMT were selected for survival analysis. To correct for baseline parameters, a multivariable Cox proportional hazard model was fitted with GMT initiation as a time-dependent variable for those with all baseline parameters available (*n* = 47). Baseline characteristics were evaluated from 2 months prior or a maximal of 2 days after the MTB in which patients received their recommendation.

When more than one GMT was initiated, the therapy with best overall duration on treatment was selected. Best radiographic and PSA responses were assessed according to the response evaluation criteria in solid tumours (RECIST1.1) [41] and prostate cancer clinical trials working group (PCWG3) criteria [42], respectively. Univariable and multivariable competing risk analyses were used to identify parameters associated with the initiation of a GMT [43]. A *P* value < 0.05 was considered significant for all analyses. Data were visualised using R and RStudio (v1.1.463). Other statistical tests were performed in SPSS (v25).

## Results

### Patient characteristics

**Fig. 1:**
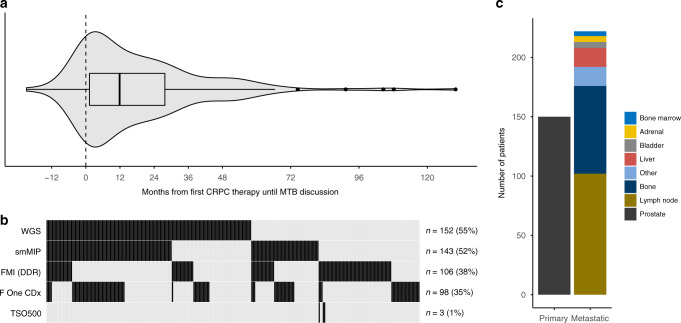
Baseline patient characteristics. **a** Violin plot and boxplot visualising time from first castration-resistant prostate cancer (CRPC) therapy until first molecular tumour board (MTB) discussion. The boxplot presents the quartiles. **b** Gene panels for the 277 sequenced patients. *n* number of patients, F One CDx Foundation One CDx, smMIP single-molecule molecular inversion probes [37], FMI (DDR) Foundation DDRm core gene panel, TSO500 TruSight Oncology 500 [38], WGS whole-genome sequencing. TSO500 and smMIP panels are performed in-house, Foundation One CDx and DDRm core gene panel externally by Foundation Medicine and WGS by Centre for Personalised Cancer Treatment. **c** Stacked bar chart presenting the most commonly sequenced sites.

During the 3-year period, 277 PCa patients received next generation or whole-genome sequencing (WGS) results. All but three patients were castration-resistant at the time of analysis. At initial diagnosis, 81.5% had at least a T3 tumour and 57% was de novo metastatic. In this cohort, patients received their first life-prolonging therapy in the castration-resistant state a median 3.7 years after initial diagnosis (95% confidence interval [CI] 3.1–4.4 years). Of the 277 patients with sequencing results, 215 (78%) were referred to and discussed within the MTB of the Radboudumc. The median time between the first line of systemic treatment for CRPC and initial discussion at the MTB was 1 year (Fig. 1a), with a median follow-up time after first MTB discussion of 1.3 years. Almost 80% of the discussed patients received a next-generation hormonal agent and 67% taxanes (Supplementary Table A.1). At the first discussion, the median number of systemic treatment lines for CRPC was two. More than half of the patients (*n* = 152) underwent WGS as the widest gene panel with the most frequently biopsied site being the prostate (Fig. 1b, c). The most common identified pathogenically altered genes in these patients, having received sequencing on hormone-sensitive tissue and/or castration-resistant metastases, with varying panel compositions, were *TP53* (33%), *PTEN* (28%) and *AR* (18%), followed by *BRCA2* (9%). Overall survival (OS) from the start of the first CRPC therapy for the complete cohort was 3.5 years (95% CI 3.1–3.9 years).

### MTB results

**Fig. 2:**
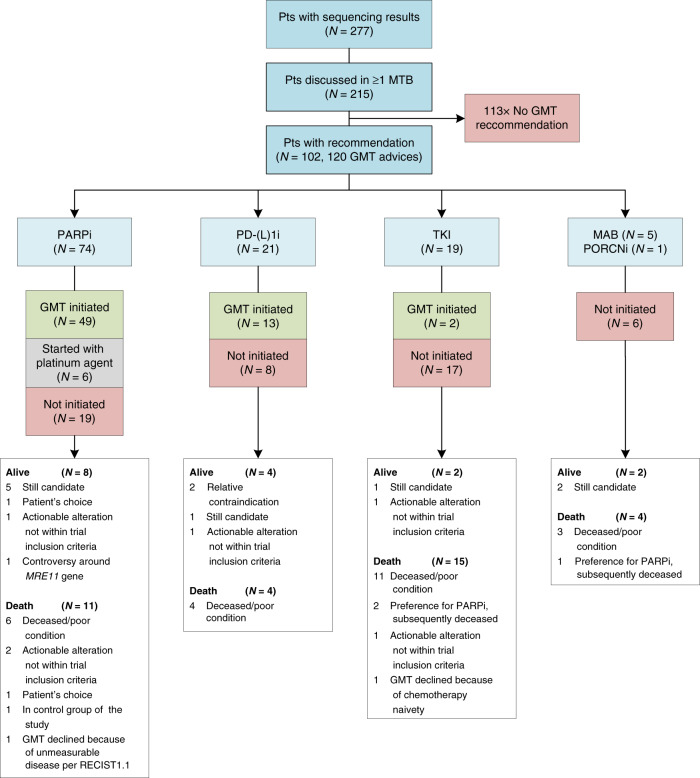
Flowchart of patients receiving sequencing results and/or discussed in Molecular Tumour board (MTB). GMT genetically matched therapy, MAB monoclonal antibodies, PARPi PARP inhibitors, PD-(L)1i programmed death(-ligand) 1 inhibitors, PORCNi porcupine inhibitors, TKI tyrosine kinase inhibitors.

**Fig. 3:**
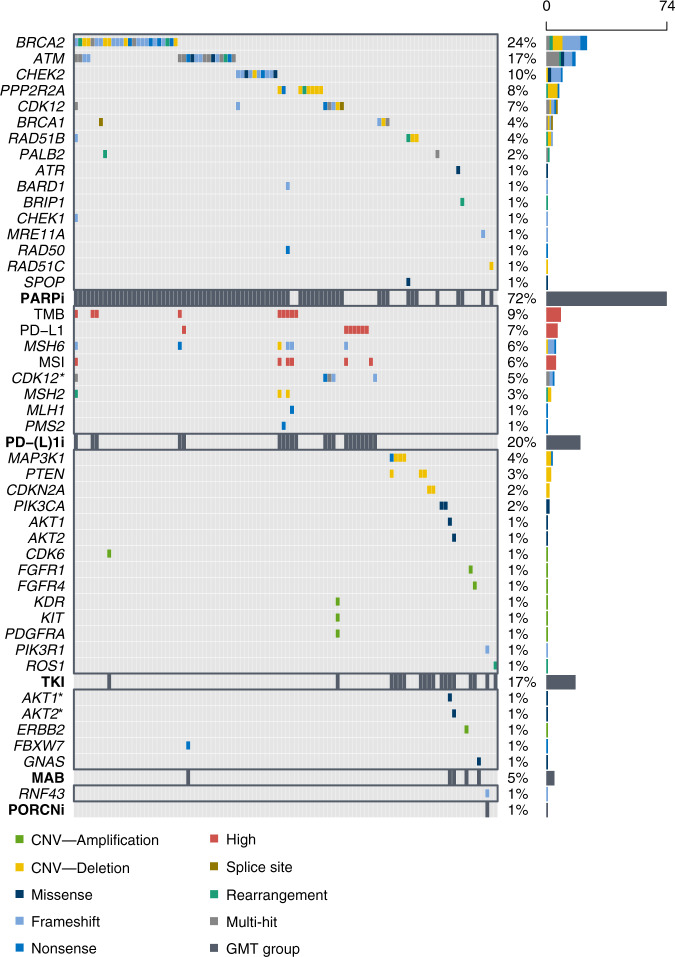
Oncoplot showing the genetic alterations and other qualifying criteria leading to the grouped genetically matched therapy (GMT) recommendations in bold. The colour of the boxes represents the effect of the alteration or the presence of a high tumour mutational burden (TMB) per inclusion criteria, microsatellite instability (MSI) or immunohistochemical programmed death-ligand 1 (PD-L1) expression ≥1%. An asterisk indicates that the gene is mentioned a second time in the figure, as it forms a rationale for two different therapies. Two or more different alterations in the same gene of the same patient is indicated by Multi Hit. Loss of the wild-type allele in case of heterozygous mutation was often not reported. CNV copy number variant, PARPi PARP inhibitors, PD-(L)1i programmed death(-ligand) 1 inhibitors, PORCNi porcupine inhibitors, TKI tyrosine kinase inhibitors.

Of the 215 PCa patients with NGS results that had been referred to and discussed within the MTB, 102 (47%) received at least one GMT recommendation (range 1–2, total: 120 recommendations; Fig. 2). PARPi were the most proposed targeted therapies with 74 recommendations (62%), followed by PD-(L)1i with 21 recommendations (18%) and Tyrosine Kinase Inhibitors (TKI) with 19 (16%). The genetic aberrations that formed the basis for the recommendations are visualised in Fig. 3. The presence of putative immunogenic genomic alterations included a high tumour mutational burden (hTMB) and microsatellite instability (MSI). In the first year of the study inclusion, PD-L1 expression ≥1% on PCa cells by immunohistochemistry was also a qualifying criterium for immunotherapy eligibility, and nine patients received PD-(L)1i based on PD-L1 expression alone. One hundred thirteen discussed patients (53%) did not receive any GMT recommendation, however in retrospect three patients did carry a potentially actionable aberration. One harboured an *ATM* mutation with a second cancer of pancreatic origin; one had a *CDK12* mutation but also a second cancer of the liver; the third was initially considered microsatellite stable (by WGS), but following additional analyses on different tissue re-evaluated to be MSI (by both immunohistochemistry and NGS). No MTB recommendation was given of the latter result. Nonetheless, this patient eventually did start treatment with a PD-(L)1i. Even though the majority of patients with NGS results were discussed within the MTB, still 62 patients were not discussed. The vast majority of these patients had no druggable mutations by screening with the Foundation One DDRm core gene panel. Five had actionable alterations and two of these patients started a GMT.

Sixty-three out of the 102 patients (62%) initiated treatment with their recommended GMT. This included six patients with DNA damage repair alterations who started platinum-based chemotherapy as a substitution for PARPi. The realisation of treatment initiation per MTB recommendation was highest for PARPi (74%), followed by PD-(L)1i (62%). Only two of the remaining 25 recommendations (8%) were implemented. The main reason for not following through on proposed advice was deterioration of performance status or (cancer-related) death (*n* = 24, 48%, Supplementary Fig. A.1). In six cases, the MTB recommendation could not be executed due to a called pathogenic gene alteration that eventually did not match the molecular trial inclusion criteria.

Patient characteristics at time of MTB discussion for those with a GMT recommendation are listed in Supplementary Table A.2. Patients that did not (yet) initiate their recommended therapy are further subdivided into a subgroup of patients that will never initiate GMT due to death in follow-up. A significant shorter follow-up time from recommendation was seen in patients who did not (yet) initiate their recommended therapy (total and deceased subgroup). Those that initiated a GMT, showed a lower alkaline phosphatase level but comparable other prognostic variables to patients that did not (yet) initiate GMT. Of these parameters, we assessed their predictive value on starting a recommended GMT in a competing risk analysis with death as competing risk (Table 1). A longer time between first CRPC therapy and advice, more than two treatment lines for CRPC, the presence of liver metastasis and a higher PSA all were associated with increased odds of initiating the recommended GMT. When adjusting for which GMT was recommended in order to mitigate confounding, PSA and the time between first CRPC therapy and advice were no longer significantly associated, while lower haemoglobin was significantly associated with initiating a recommended GMT (Table 1). The presence of liver or any visceral metastasis, higher lactate dehydrogenase, higher neutrophil-to-lymphocyte ratio, lower albumin, and lower haemoglobin were significantly associated with shorter OS.

**Table 1:** Competing risk analysis.

		Started		Started, adjusted		Death	
	*n*	HR	*P* value	HR	*P* value	HR	*P* value
Age at recommendation	101	0.9886	0.3920	0.9834	0.2464	1.0349	0.1260
Months from CRPC to recommendation	101	1.0145	**0.0116**	1.0110	0.0625	1.0065	0.4531
Treatment lines >2	101	2.0957	**0.0036**	1.8137	**0.0219**	1.4944	0.3266
Presence of visceral metastasis	101	1.2824	0.3381	1.5035	0.1189	2.3309	**0.0317**
Presence of liver metastasis	101	2.7837	**0.0003**	2.6695	**0.0003**	5.4456	**0.0029**
Laboratory parameters at the time of recommendation							
Prostate-specific antigen	90	1.0002	**0.0242**	1.0002	0.0971	1.0006	0.2190
Lactate dehydrogenase	84	1.0009	0.5247	1.0020	0.1050	1.0044	**<0.0001**
Alkaline phosphatase	86	0.9999	0.3544	0.9999	0.6121	1.0001	0.8238
Haemoglobin	92	0.7917	0.1145	0.7170	**0.0380**	0.4488	**<0.0001**
Albumin	79	0.9621	0.4376	1.0052	0.9216	0.8633	**0.0239**
Neutrophil-to-lymphocyte ratio	57	0.9851	0.7745	0.9872	0.8051	1.1901	**0.0005**

*CRPC* castration-resistant prostate cancer, *HR* hazard ratio, *n* number of patients in the analysis.

Univariable competing risk analysis identifying variables associated with starting the recommended therapy (started) or death. A multivariable analysis adjusting the variables for the confounder recommended therapy is presented in started, adjusted. Study subject 061 is excluded for analysis since he was discussed after his death. Significant *P* values are in bold.

### Clinical outcome

**Fig. 4:**
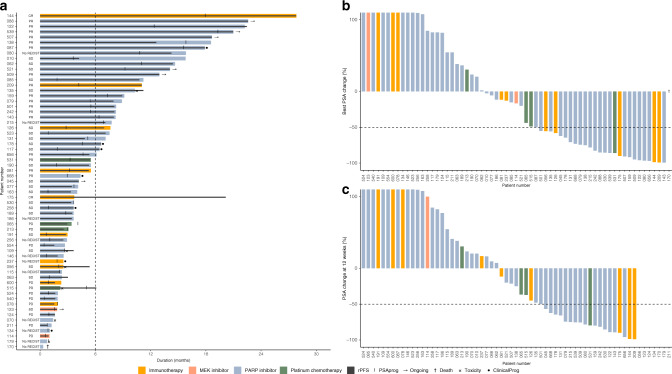
Response on genetically matched therapy (GMT). **a** Time on treatment of all 63 patients who started a GMT indicated by coloured bars. The black bar indicates radiographic or clinical progression-free survival. Termination of therapy as a result of toxicity or clinical progression is indicated accordingly. Text left to the bars indicates the best radiographic response: CR complete response, PR partial response, SD stable disease, PD progressive disease. Clinicalprog, clinical progression according to treating physician; PSAprog, increase of >25% from lowest PSA; rPFS, radiographic progression-free survival. **b**, **c** Waterfall plot of best prostate-specific antigen (PSA) response to the genetically matched therapy indicated by colour scheme at any time (**b**) or 12 weeks (**c**). Note, the *y* axis is cut-off at +100%.

The median time on treatment for the 63 patients who started a GMT was 5.5 months (95% CI 3.65–7.66, Fig. 4a and Supplementary Fig. A.2). Median PFS on GMT was 5.3 months (95% CI 3.5–7.1, Supplementary Fig. A.2). For both PARPi and PD-(L)1i specifically, this was 5.3 months. For platinum-based chemotherapies, a median PFS of 4.2 months was reached. Twenty-six of the 63 patients (41.3%) showed a PFS ≥ 6 months. Median biochemical PFS was 3.6 months (95% CI 2.8–5.5, Supplementary Fig. A.2). A PSA decline ≥50% was witnessed in 27 patients (43.5%) (Fig. 4b, c). Highest PSA declines were seen for platinum-based chemotherapies (median –46.4%, interquartile range (IQR) −76.7 to –11.8%), followed by PD-(L)1i (median −34.1%, IQR −92.2 to −158.3%) and PARPi (median −13.4%, IQR −79.3 to –82.4%). Out of the 52 patients that could be evaluated for response per RECIST1.1 criteria, 20 (38.5%) showed an objective radiographic response, either partial (*n* = 18) or complete response (*n* = 2, both PD-L1i). Twenty-two (42.3%) patients had stable disease as the best response, and ten patients (19.2%) had progressive disease. Minimal differences were seen in objective response rates between therapies (PARPi, 37.8%; PD-L1i, 44.2%; platinum-based chemotherapies, 50%).

Median OS from the time of GMT recommendation was 19.0 months (IQR 14.7–23.2) for the subgroup of patients initiating the GMT. From the first CRPC therapy, median OS was 47.8 months (IQR 35.0–60.7). For the subgroup in which the recommendation could not be followed, the median OS was only 5.9 months (IQR 2.5–9.4) from recommendation and 27.0 months (IQR 18.6–35.5) from first CRPC therapy. The median OS from the first CRPC therapy for the patients who did not harbour an actionable aberration was 39.2 months (IQR 34.4–44.0). Since performance status was the main reason for not initiating a recommended GMT, we corrected OS from time of recommendation for known prognostic laboratory parameters and the presence of liver metastasis at the time of recommendation for patients with all parameters available (*n* = 47). In this multivariable analysis, initiation of a GMT appeared most significantly associated with a longer OS from time of recommendation (hazard ratio 0.626, 95% CI 0.527–0.742, *P* < 0.001; Supplementary Table A.3).

### ESMO precision medicine working group recommendations

Finally, we re-assessed the actionability of all pathogenic aberrations according to the 2020 ESMO Precision Medicine Working Group recommendations for NGS in advanced prostate cancer [44]. This showed at least one actionable genomic alteration in 126 of the 277 (45.5%) patients, agnostic of the panel size (Supplementary Fig. A.3). As gene panel composition varied, we evaluated the prevalence of key druggable genes such as *PTEN*, *AKT* and *PIKC3*, *BRCA1* and *BRCA2*, and MSI in the tested population; 35% harboured at least one pathogenic alteration in *PTEN*, 2% in both *AKT1* and *PIK3CA*, and 2% and 10% in *BRCA1* and *BRCA2*, respectively. MSI was present in 4% of the tested cases.

## Discussion

In this retrospective study, we describe the experience and results of ﻿genomics-driven, patient-tailored treatment recommendations of our molecular tumour board (MTB) in a tertiary cancer referral centre with specific expertise in prostate cancer in the south–east of the Netherlands. By evaluating the data of 215 PCa patients discussed in the MTB, this study is among the largest studies describing the impact of an MTB on routine clinical cancer care and trial allocation, and to our knowledge the largest specifically focusing on PCa. Almost half of the 215 discussed patients received a GMT recommendation, with >60% initiating the therapy as suggested by the MTB. Clinical benefit was seen across GMT subclasses of PARPi, platinum-based chemotherapy and PD-(L)1i, with overall a median PFS of 5.3 months on GMT and 41.3% of patients showing a PFS ≥ 6 months. Multivariable analyses suggested a contribution of GMT initiation on overall survival in those patients harbouring an actionable alteration when corrected for known prognostic variables.

In comparison to other MTBs that have reported recommendations in solid tumours, the proportion of PCa patients with GMT allocation by this MTB is relatively high. In the study of Basse et al., with the second-most PCa patients included, only 7% of the discussed PCa patients started a GMT [9] and in the ProfiLER trial only 3 of the 53 genito-urinary cancer patients started [17]. Other MTB studies with more than 100 study subjects all reached a GMT implementation rate of less than the circa 30% reached in this study [7, 8, 10–12, 14, 16]. This implies routine sequencing can greatly impact GMT allocation in CRPC patients. Note should be taken that in more than half of the patients (in particular those who received WGS), fresh biopsies of metastatic tissue sites were taken, in comparison with many reports utilising archival primary tissue. Also, these data are likely prone to physician-based selection bias, since patients with an intriguing course of disease (such as no response to androgen deprivation therapy or chemotherapy), patients with a positive family history or patients with younger age at disease manifestation were more likely to undergo NGS and be referred for MTB discussion. These and other factors in part may explain differences with previous publications.

The prospective MOSCATO-01 trial and the randomised controlled Phase 2 SHIVA trial (trials evaluating the clinical benefit of GMTs in advanced cancers), reported a PFS on targeted therapy of 2.3 and 2.0 months, respectively [45, 46], which is remarkably inferior to the 5.3 months observed in this study for PCa. Benefit from the principal actionable targets PARPi and checkpoint immunotherapies, which made up a large proportion of GMTs, might therefore be greater than the targeted agents used in other precision-oncology trials for solid tumours. Olaparib received recent EMA registration for *BRCA1* and *BRCA2* mutated PCa [47], showing a median PFS of 9.8 months in this molecular subtype [48]. The full scope of the effect of PARPi on non-*BRCA* DNA damage repair-deficient mCRPC patients is to be elucidated. While alterations in non-*BRCA* genes show limited antitumour activity to PARPi, these analyses are commonly hampered by low numbers. Nevertheless, *PALB2, RAD51B, CDK12* and less frequently *ATM*, are examples of genes with response to PARPi beyond *BRCA* [25, 49]. In our cohort, most patients received PARPi in the scope of clinical trials. This led to patients receiving PARPi based on gene alterations that were thought to predict response, but currently do not fit the label, or in hindsight were genes without any efficacy to PARPi, particularly for *PPP2R2A* [50]. Outcomes to PARPi might even be greater with a more narrow patient selection. Checkpoint immunotherapies in molecularly selected PCa populations, including homologous recombination repair deficiency, hTMB and MSI, show enhanced responsiveness when compared to unselected subtypes [30, 51, 52]. The treatment period might also contribute to the superior results from this study when compared to the MOSCATO and SHIVA trials [53].

There appears to be a survival benefit in patients who initiated a GMT after correcting for known prognostic differences. We acknowledge that these analyses are still prone to bias, as performance status was one of the main reasons for receiving precision medicine or any other line of therapy. Nevertheless, a median OS from the castration-resistant state of 4 years for our patient cohort is notable, considering a comparable Dutch mCRPC population showed a median survival of less than 3 years from castration resistance [23]. These data suggest a survival benefit in settings with access to comprehensive NGS panels, and a high probability of genetic matching of actionable alterations to a corresponding precision-oncology programme.

Still, half of all discussed patients did not receive a recommendation for a GMT, for which de facto the most contributing factor remains a lack of drugs to match identified gene defects. Also, a limited number of early-phase cancer trials with targeted agents in the Radboudumc, were specifically for prostate cancer or cancer type agnostic. The variety of sequencing methods and the relatively limited size of targeted gene panels also possibly contributed. About half the cohort was analysed with targeted sequencing only, while whole-genome/exome sequencing enlarges the probability of finding druggable aberrations [54, 55]. Although, in our data, the proportion of patients without recommendation was roughly the same for the WGS and targeted-sequencing patients. Targeted panels were often performed in academic setting, or in the scope of Phase 2 or 3 biomarker-driven clinical trials, while WGS was commonly utilised for more explorative personalised medicine trials such as the DRUP trial (NCT02925234). Aggressive variant prostate cancer, molecularly assessed by combinatory alterations in *RB1*, *TP53* and *PTEN*, was not considered druggable in our study. Following the results of the Phase 1–2 trial by Corn et al. this subtype of patients could be considered druggable with carboplatin added to cabazitaxel, thereby further expanding the proportion of GMT-susceptible CRPC patients [56].

The 62 patients who were not discussed were predominantly patients with a negative NGS result, probably leading to an overestimation of the proportion of MTB-discussed patients with a recommendation for a GMT. Patients with only targeted sequencing were to be actively referred for MTB discussion. In the case of none reported alterations within small targeted-sequencing panels, it happened that patients were not referred for discussion.

For 40% of the patients receiving a GMT recommendation, the advice was not followed up by the treating oncologist. The main reason for not initiating a GMT was a deterioration in performance status or death after molecular profiling. Other studies identified this issue similarly as the greatest bottleneck [6, 9, 16]. Through competing risk analyses with death as competing risk, corrected for GMT recommendation, we identified lower haemoglobin, presence of liver metastasis and more than two treatment lines as predictive factors for initiation of a GMT. The number of treatment lines and the presence of liver metastasis can be explained by inclusion criteria for the DRUP trial (NCT02925234), as it is mandatory to have had at least two lines of systemic treatment and measurable disease. Lower haemoglobin is a poor prognostic feature also associated with previous chemotherapy and diffuse bone metastases, with levels below the lower limit of normal being a common exclusion criterium for clinical trials. Low haemoglobin might have led to more urge of initiating a GMT. Low haemoglobin was also significantly associated with outcome in the same analysis, as was the presence of visceral or liver metastasis, higher lactate dehydrogenase or neutrophil-to-lymphocyte ratio and lower albumin. This is in line with the Royal Marsden Hospital prognostic score including albumin, lactate dehydrogenase and the number of metastatic sites, and with an mCRPC-specific prognostic score, incorporating also liver metastasis, haemoglobin, alkaline phosphatase and the neutrophil-to-lymphocyte ratio [57, 58].

A point of debate remains the optimal timing for referral of a PCa patient to an MTB. Both the European Society for Medical Oncology (ESMO) and the National Comprehensive Cancer Network (NCCN) provide recommendations for genetic testing in PCa patients [59, 60]. Just over half of the panellists at the 2019 Advanced Prostate Cancer Consensus Conference voted for genetic testing at diagnosis of metastatic disease [61]. Our strategy is to test PCa patients predominantly early in the metastatic state since the genomics of CRPC might differ from nonlethal primary PCa [62]. As the cost of molecular analysis is only 6% of the total cost of targeted therapy and MTB referral is just 0.3%, genetic profiling and prompt MTB discussion should be feasible for every CRPC patient from a physician’s standpoint [63]. The cost-effectiveness of early routine molecular characterisation leading to the possible implementation of GMT is being analysed by the PROMPT study (NCT04746300), also evaluating the effectiveness and patient-reported quality of life outcomes.

This study should be viewed in the context of several limitations. The MTB provided recommendations based on trials and insights at the moment of discussion, leading to an inconsistency between genetic aberrations and their matched therapies over the 3 years of inclusion. The retrospective nature of this study may have biased the interpretation of a small number of inconclusive MTB reports. The use of gene panels with different sizes and focus led to underreporting of several putative actionable genes. The usage of one broad NGS gene panel on one platform would streamline molecular profiling and give a better representation of actionable molecular landscape of CRPC. The heterogenous patient population, especially regarding previous treatment lines, makes the clinical benefit of GMT in this study hard to interpret. Therefore, the PROMPT study uses one NGS platform to profile patients in the same treatment line.

## Conclusion

This study analysed NGS data of 277 PCa patients of which a majority had been referred to and discussed within an MTB. Almost half the patients received a targeted or immunotherapy recommendation, of which in 62% the recommended therapy was initiated. Median PFS following initiation of GMT was 5.3 months, with 41.3% of patients demonstrating a PFS longer than 6 months. Our data indicate that the underrepresentation of PCa patients in MTB discussions is unjustified in comprehensive cancer centres with broad access to precision-oncology programmes. In the current era of precision oncology for PCa, our aim should be to be able to characterise the genetic background of every CRPC patient for them to fully profit from GMTs in routine use and within clinical trials. Future studies should include value-based health care endpoints as costs for complex molecular diagnostics and MTB discussion are not yet reimbursed by health insurance companies.

## Supplementary information

Appendix 1

Appendix 2

## Data Availability

The datasets generated during and/or analysed during this study are available in the DANS Easy repository, 10.17026/dans-zkk-m76n.
