# Correction to: Impact of molecular tumour board discussion on targeted therapy allocation in advanced prostate cancer

**DOI:** 10.1038/s41416-022-01765-y

**Published:** 2022-02-24

**Authors:** Peter H. J. Slootbeek, Iris S. H. Kloots, Minke Smits, Inge M. van Oort, Winald R. Gerritsen, Jack A. Schalken, Marjolijn J. L. Ligtenberg, Katrien Grünberg, Leonie I. Kroeze, Haiko J. Bloemendal, Niven Mehra

**Affiliations:** 1 Radboud University Medical Centre, Radboud Institute for Health Sciences, Department of Medical Oncology, Nijmegen, The Netherlands; 2 Radboud University Medical Centre, Radboud institute for Molecular Life sciences, Department of Experimental Urology, Nijmegen, The Netherlands; 3 Radboud University Medical Centre, Radboud Institute for Health Sciences, Department of Urology, Nijmegen, The Netherlands; 4 Radboud University Medical Centre, Radboud Institute for Molecular Life sciences, Department of Pathology, Nijmegen, The Netherlands; 5 Radboud University Medical Centre, Radboud Institute for Molecular Life sciences, Department of Human Genetics, Nijmegen, The Netherlands

**Keywords:** Prostate cancer, Cancer genetics, Targeted therapies, Cancer immunotherapy

Correction to: *British Journal of Cancer* 10.1038/s41416-021-01663-9, published online 15 December 2021

The article "Impact of molecular tumour board discussion on targeted therapy allocation in advanced prostate cancer ", written by Peter H. J. Slootbeek, Iris S. H. Kloots, Minke Smits, Inge M. Oort, Winald R. Gerritsen, Jack A. Schalken, Marjolijn J. L. Ligtenberg, Katrien Grünberg, Leonie I. Kroeze, Haiko J. Bloemendal, Niven Mehra, was originally published electronically on the publisher’s internet portal on 15 December 2021 without open access. With the author(s)’ decision to opt for Open Choice the copyright of the article changed on 13 January 2022 to © The Author(s) 2022 and the article is forthwith distributed under a Creative Commons Attribution 4.0 International License, which permits use, sharing, adaptation, distribution and reproduction in any medium or format, as long as you give appropriate credit to the original author(s) and the source, provide a link to the Creative Commons licence, and indicate if changes were made. The images or other third party material in this article are included in the article’s Creative Commons licence, unless indicated otherwise in a credit line to the material. If material is not included in the article’s Creative Commons licence and your intended use is not permitted by statutory regulation or exceeds the permitted use, you will need to obtain permission directly from the copyright holder. To view a copy of this licence, visit http://creativecommons.org/licenses/by/4.0/.

