# Supplementary material for: Impact of molecular tumour board discussion on targeted therapy allocation in advanced prostate cancer
Source: Br J Cancer. 2021 Dec 15;126(6):907–16. doi: 10.1038/s41416-021-01663-9 (PMC8927341; doi:10.1038/s41416-021-01663-9)
Supplement: Supplementary file 1 — Appendix 1 [file 41416_2021_1663_MOESM1_ESM.pdf]

Appendix to:

Impact of molecular tumour board discussion on targeted therapy allocation in advanced prostate cancer

Peter H. J. Slootbeek, Iris S.H. Kloots, M. Smits, Inge M. van Oort, Winald R. Gerritsen, Jack A. Schalken, Marjolijn J.L. Ligtenberg, Katrien Grünberg, Leonie I. Kroeze, Haiko J. Bloemendal, Niven Mehra

Tables of contents:

Page 2 **Table A.1** Therapies before initial MTB discussion

Page 3 **Table A.2** Patient characteristics at time of MTB discussion for those with a GMT recommendation

Page 4 **Table A.3** Results of a multivariable analysis of overall survival from recommendation.

Page 5 **Figure A.1** Reasons for not initiating a recommended genetically matched therapy. MAB, monoclonal antibodies; PARPi, PARP inhibitors; PD-(L)1i, Programmed Death(-Ligand) 1 inhibitors; PORCNi, Porcupine inhibitors; SOC, standard of care; TKI, Tyrosine Kinase Inhibitors.

Page 6 **Figure A.2** Kaplan-Meier curves visualizing time on treatment on a genetically matched therapy (upper), radiographic or clinical progression free survival (PFS) (middle) and biochemical PFS (lower). The grey area indicates the 95% confidence interval and the dotted line the median time.

Page 7 **Figure A.3** Oncoplot showing the pathogenic or likely pathogenic alterations or presence of the actionable targets according to the 2020 ESMO Precision Medicine Working Group recommendations for NGS in advanced prostate cancer. Two or more alterations in the same gene of the same patient is indicated by Multi Hit. CNV, copy number variant.

Table A.1 Therapies before initial MTB discussion

Lines of systemic therapies targeting CRPC	Number of patients (%)
0	22 (10.4)
1	74 (34.4)
2	47 (21.9)
3	32 (14.9)
4	21 (9.8)
5	13 (6.0)
6	6 (2.8)
Types of systemic therapies targeting CRPC	
Next-generation hormonal agents	170 (79.1)
Enzalutamide	106 (49.3)
Abiraterone	109 (50.7)
Taxane chemotherapies	144 (67.0)
Docetaxel	144 (67.0)
Upfront	62 (22.4)
Cabazitaxel	38 (17.7)
Radiopharmaceuticals	47 (21.9)
Radium-223	39 (18.1)
PSMA-radioligand therapy	14 (6.5)

The number of systemic therapy lines and most commonly initiated systemic therapies aimed at CRPC before initial MTB discussion, for all 215 patients discussed in at least one MTB meeting. CRPC, castration-resistant prostate cancer; MTB, molecular tumour board; PSMA, prostate-specific membrane antigen.

**Table A.2** Characteristics at time of recommendation		All patients with MTB recommendation	Started		Not started, total			Not started, deceased		
	n	No. of patients (valid %) Median [IQR]	n	No. of patients (valid %) Median [IQR]	n	No. of patients (valid %) Median [IQR]	*P*-value	n	No. of patients (valid %) Median [IQR]	*P*-value
Age at MTB of advice, years	101	69.0 [62.6-74.7]	63	67.2 [61.6-73.4]	38	70.7 [63.7-75.3]	0.112	26	70.8 [64.6-75.0]	0.140
Time from first CRPC therapy to recommendation, years	101	1.4 [0.5-2.5]	63	1.8 [0.9-2.7]	38	0.8 [0.2-2.0]	**0.008**	26	1.3 [0.5-2.0]	0.176
Follow-up from MTB of advice, years	101	1.4 [0.5-2.5]	63	1.5 [0.8-2.1]	38	1.0 [0.3-1.9]	**0.033**	26	0.5 [0.2-1.3]	**<0.001**
Lines of CRPC therapies before initial MTB discussion	101		63		38			26		
0-1		38 (37.6)		21 (33.3)		17 (44.7)			8 (30.8)	
2		23 (22.8)		12 (19.0)		11 (28.9)	0.102	26	9 (34.6)	0.312
≥ 3		40 (39.6)		30 (47.6)		10 (26.3)			9 (34.6)	
Presence of visceral metastasis	101	34 (33.7)	63	21 (33.3)	38	13 (34.2)	0.741	26	11 (42.3)	0.846
Presence of Liver metastasis	101	17 (16.8)	63	12 (19.0)	38	5 (13.2)	0.165	26	5 (19.2)	0.333
PSA, µg/l	90	77.5 [18.8-255.0]	57	74.0 [15.5-215.0]	33	110.0 [22.8-300.0]	0.469	24	172.4 [47.0-310.0]	0.066
LDH, U/l	84	230.0 [202.5-285.8]	52	222.0 [202.5-574.3]	32	241.0 [204.8-377.5]	0.458	21	245.0 [213.5-417.0]	0.172
ALP, U/l	86	115.0 [87.8-203.5	53	102.0 [85.0-168.0]	33	132.0 [108.0-251.0]	**0.027**	22	182.0 [118.8-289.8]	**0.002**
HB, mmol/l	92	7.5 [6.8-8.1]	55	7.5 [6.8-8.1]	37	7.4 [6.8-8.4]	0.946	25	7.0 [6.2-8.2]	0.189
Albumin, g/l	79	35.0 [32.0-37.0]	48	35.0 [33.0-37.0]	31	35.0 [32.0-37.0]	0.639	21	35.0 [32.0-36.0]	0.294
NLR	57	3.5 [2.1-5.8]	35	3.2 [2.2-5.3]	22	4.8 [2.0-7.4]	0.451	18	5.4 [2.1-7.7]	0.087

Not started, total contains all patients who have not initiated their recommended GMT and were suitable for this analysis. Not started, deceased only includes those who did not initiate their recommended GMT and are deceased. Both subgroups were compared to the subgroup who initiated their recommended GMT (Started). Study subject 061 is excluded for analysis since he was discussed after his death. ALP, alkaline phosphatase; CRPC, castration-resistant prostate cancer; HB, hemoglobin; IQR, interquartile range; LDH, lactate dehydrogenase; MTB, molecular tumour board; NLR, neutrophil to lymphocyte ratio; PSA, prostate specific antigen.

**Table A.3.a** Variables in equation			
Variable	*P*-value	HR	95%CI
Started GMT	<0.001	0.626	0.572 - 0.742
Presence of liver metastasis	0.002	3.764	1.627 - 8.707
Albumin	0.032	0.884	0.789 - 0.990

**Table A.3.b** Variables not in equation	
Variable	*P*-value
PSA	0.180
LDH	0.350
ALP	0.989
HB	0.641
NLR	0.649

Results of a multivariable logistic regression. Liver metastasis indicates the presence of liver metastasis at time of therapy recommendation. ALP, alkaline phosphatase; CI, confidence interval; HB, haemoglobin; HR, hazard ratio; LDH, lactate dehydrogenase; NLR, neutrophil to lymphocyte ratio; PSA, prostate specific antigen

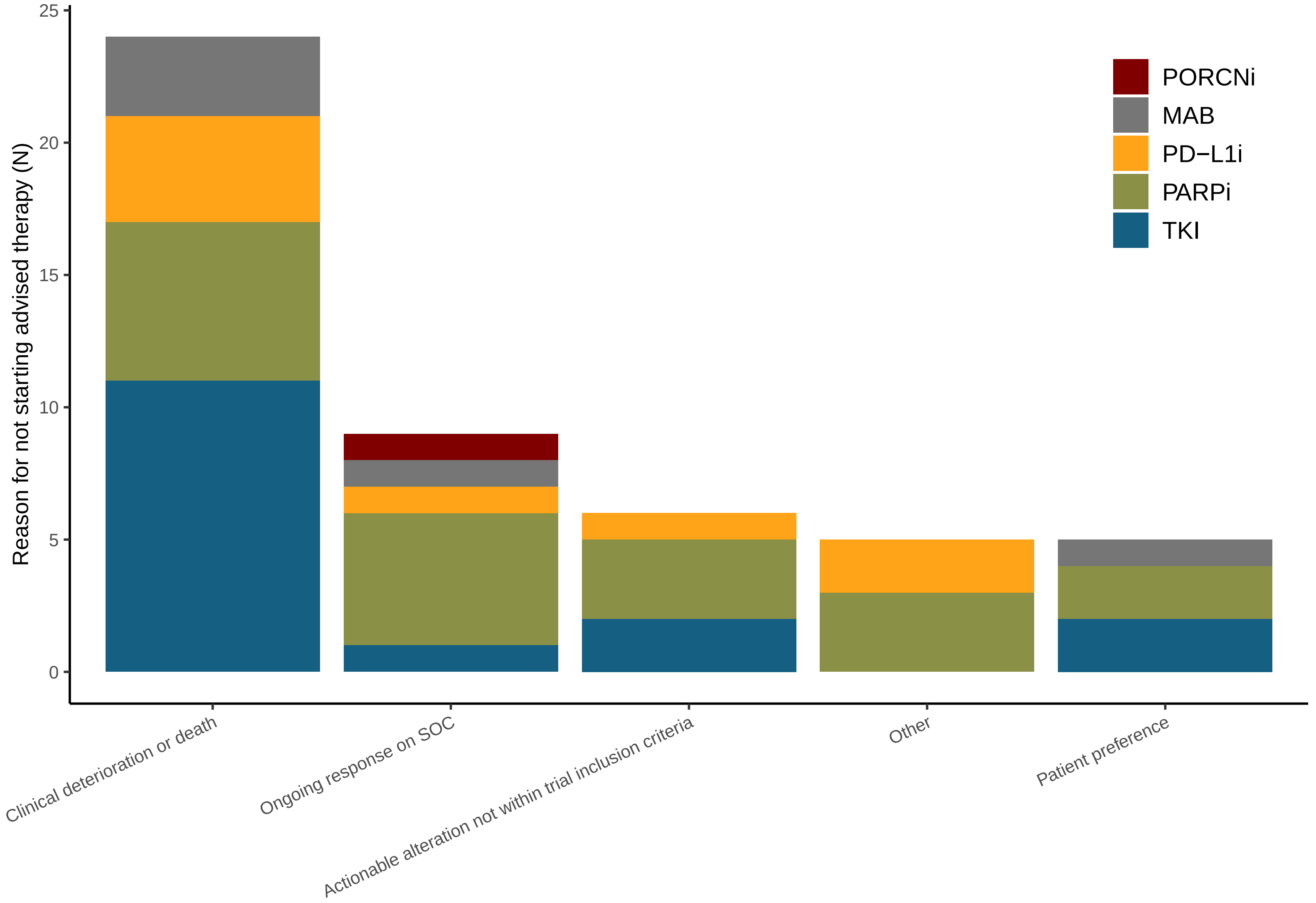

Reason for not starting advised therapy (N)
0
5
10
15
20
25
PORCNi
MAB
PD−L1i
PARPi
TKI
Clinical deterioration or death
Ongoing response on SOC
Actionable alteration not within trial inclusion criteria
Other
Patient preference

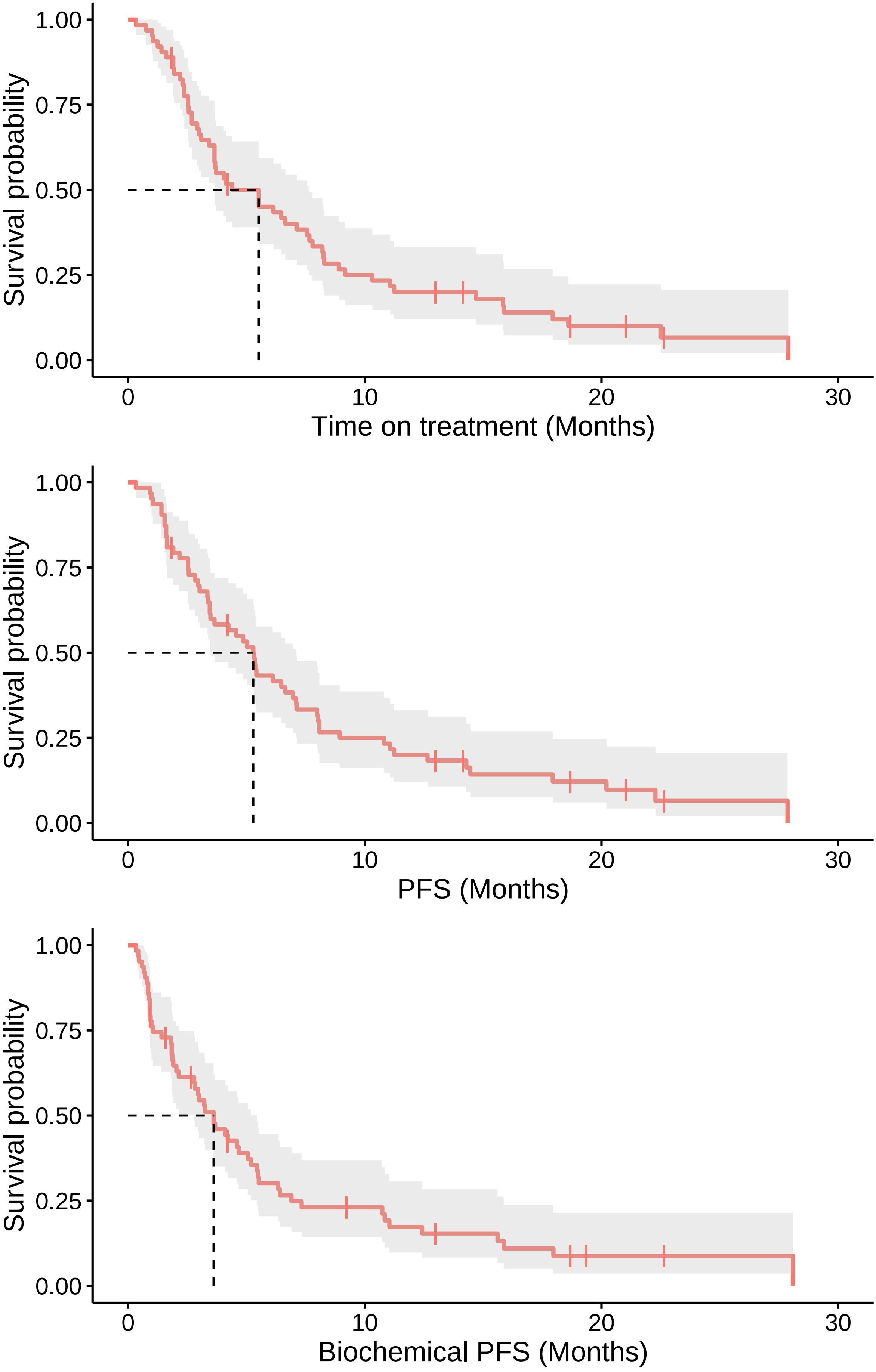

Survival probability
1.00
0.75
0.50
0.25
0.00
0
10
20
30
Time on treatment (Months)
Survival probability
1.00
0.75
0.50
0.25
0.00
0
10
20
30
PFS (Months)
Survival probability
1.00
0.75
0.50
0.25
0.00
0
10
20
30
Biochemical PFS (Months)

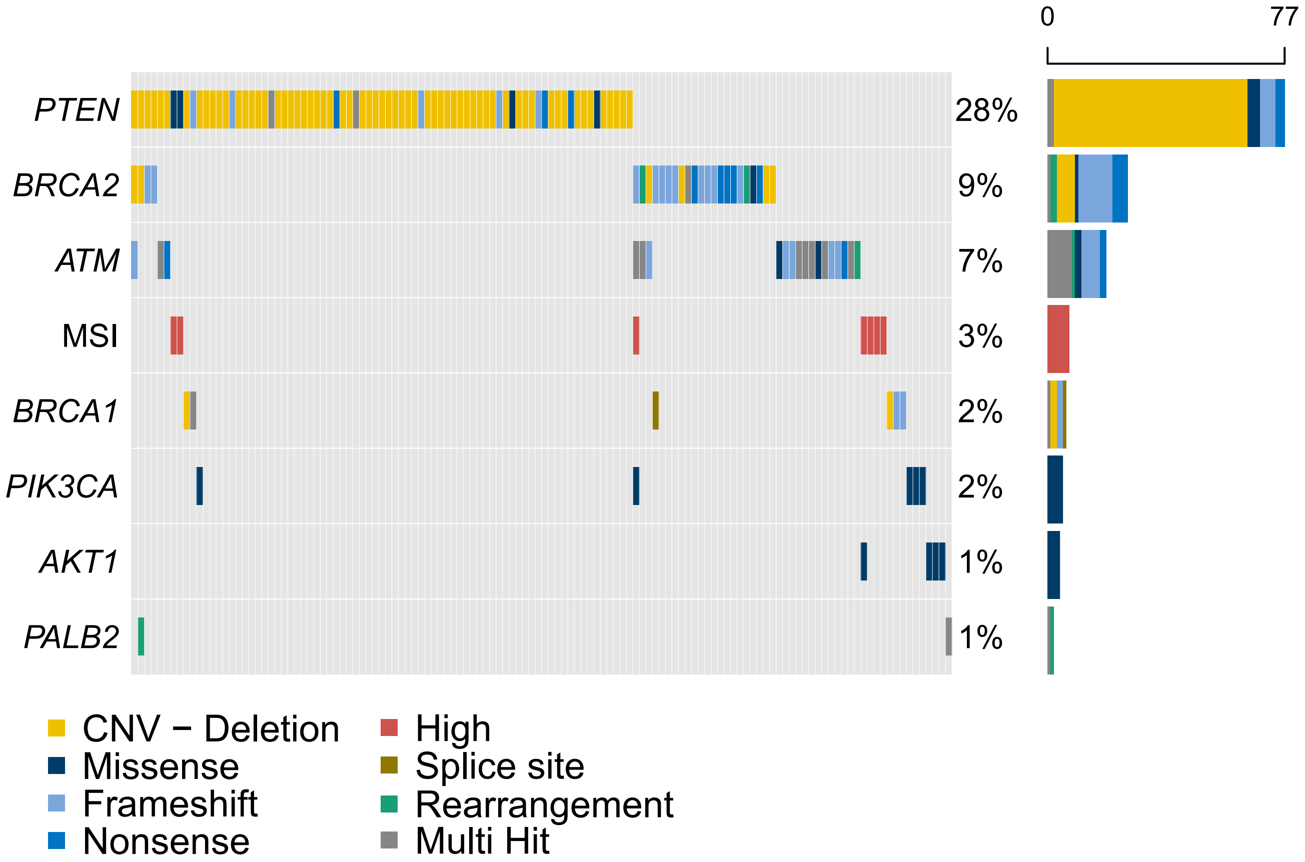
0
77
PTEN
BRCA2
ATM
MSI
BRCA1
PIK3CA
AKT1
PALB2
28%
9%
7%
3%
2%
2%
1%
1%
CNV − Deletion
Missense
Frameshift
Nonsense
High
Splice site
Rearrangement
Multi Hit
