# Supplementary material for: Impact of molecular tumour board discussion on targeted therapy allocation in advanced prostate cancer
Source: Br J Cancer. 2021 Dec 15;126(6):907–16. doi: 10.1038/s41416-021-01663-9 (PMC8927341; doi:10.1038/s41416-021-01663-9)
Supplement: Supplementary file 2 — Appendix 2 [file 41416_2021_1663_MOESM2_ESM.pdf]

Appendix 2. Gene panels used.

Since the whole genome sequencing panels and the FMI (DDR) panels were adjusted during the study period, the first used and last used or present panel are presented

Whole genome sequencing			*FMI (DDR)*			*smMIP panels*			
**First used**	**Present**	**Foundation one CDX**	**First used**	**Last used**	**TSO500**	**Radboud cancer hotspot**	**PATHv2D**	**HR-panel**	**BRCA-panel**
ABL1	ABCB1	ABL1	ATM	ATR	AR	AKT1	AKT1	BRCA1	BRCA1
AKT1	ABL1	ACVR1B	ATR	BRCA1	ATM	BRAF	AKT2	BRCA2	BRCA2
ALK	ACVR1	AKT1	BRCA1	BRCA2	B2M	CTNNB1	AKT3	BRIP1	
APC	ACVR1B	AKT2	BRCA2	CHEK2	BRAF	CXCR4	ALK	RAD51C	
ATM	ACVR2A	AKT3	CHEK1	FANCA	BRCA1	EGFR	ARAF	RAD51D	
BRAF	ADAM30	ALK	CHEK2	MLH1	BRCA2	ERBB2	BRAF		
BRCA1	AGBL4	ALOX12B	ERCC2	MRE11A	BRIP1	EZH2	BRAF		
BRCA2	AIM1	AMER1 (FAM123B)	ERCC4	NBN	CCND1	GNA11	DDR2		
CDH1	AJUBA	APC	FANCA	PALB2	CDK12	GNAQ	EGFR		
CDKN2A	AKT1	AR	FANCC	RAD51C	CDK4	GNAS	EGFR		
CSF1R	ALB	ARAF	FANCD2		CDK6	H3F3A	ERBB2		
CTNNB1	ALK	ARFRP1	FANCE		CDKN2A	H3F3B	ERBB2		
EGFR	AMBRA1	ARID1A	FANCF		ERBB2	HRAS	FGFR1		
ERBB2	AMER1	ASXL1	FANCG		ERBB4	IDH1	FGFR2		
ERBB4	ANK1	ATM	FANCI		FANCL	IDH2	FGFR3		
EZH2	ANKRD11	ATR	FANCL		FGFR1	JAK2	GNA11		
FBXW7	APC	ATRX	FANCM		FGFR2	KIT	GNAQ		
FGFR1	AR	AURKA	MLH1		FGFR3	KRAS	GNAS		
FGFR2	ARAF	AURKB	MRE11A		FGFR4	MPL	HRAS		
FGFR3	ARHGAP35	AXIN1	MSH2		HRAS	MYD88	IDH1		
FLT3	ARID1A	AXL	MSH6		JAK1	NRAS	IDH2		
GNA11	ARID1B	BAP1	MUTYH		KIT	PDGFRA	JAK2		
GNAQ	ARID2	BARD1	NBN		KRAS	PIK3CA	KIT		
GNAS	ARID5B	BCL2	NUDT1		MAP2K1	SF3B1	KIT		
HNF1A	ASXL1	BCL2L1	PALB2		MAP2K2		KRAS		
HRAS	ASXL2	BCL2L2	PARP1		MAP2K4		KRAS		
IDH1	ATG7	BCL6	PARP2		MAP3K1		MAP2K1		

IDH2	ATM	BCOR	PARP3	MET	MDM2
JAK2	ATP1A1	BCORL1	PMS2	MLH1	MET
JAK3	ATP2B3	BRAF	POLD1	MSH2	MET
KDR	ATR	BRCA1	POLE	NOTCH1	MTOR
KIT	ATRX	BRCA2	RAD50	NOTCH2	NRAS
KRAS	AXIN1	BRD4	RAD51	NOTCH3	PDGFRA
MET	AXIN2	BRIP1	RAD51B	NOTCH4	PDGRFA
MLH1	AXL	BTG1	RAD51C	NRAS	PIK3CA
MPL	B2M	BTG2	RAD51D	PALB2	PIK3CA
NOTCH1	BAP1	BTK	RAD52	PDGFRA	POLE
NPM1	BARD1	C11orf30 (EMSY)	RAD54L	PDGFRB	PTEN
NRAS	BCL2	C17orf39 (GID4)	RPA1	POLE	RAF1
PDGFRA	BCL9L	CALR	TP53BP1	PPP2R2A	ROS1
PIK3CA	BCOR	CARD11	XRCC2	PTEN	
PTEN	BIRC3	CASP8	XRCC3	RAD51B	
PTPN11	BMPR1A	CBFB		RAD51C	
RB1	BMPR2	CBL		RAD51D	
RET	BRAF	CCND1		RAD54L	
SMAD4	BRCA1	CCND2		RAF1	
SMARCB1	BRCA2	CCND3		TP53	
SMO	BRD7	CCNE1		ALK	
SRC	BRIP1	CD22		BARD1	
STK11	BTK	CD274 (PD-L1)		EGFR	
TP53	C21orf91	CD70		JAK2	
VHL	CACNA1D	CD79A		MSH6	
	CALR	CD79B		PIK3CA	
	CARD11	CDC73		RET	
	CASP8	CDH1			
	CASZ1	CDK12			
	CBFB	CDK4			
	CBL	CDK6			
	CBLB	CDK8			
	CCND1	CDKN1A			

CCND2	CDKN1B
CCND3	CDKN2A
CCNE1	CDKN2B
CCSER1	CDKN2C
CD274	CEBPA
CD44	CHEK1
CD58	CHEK2
CD79A	CIC
CD79B	CREBBP
CDC73	CRKL
CDH1	CSF1R
CDH10	CSF3R
CDK12	CTCF
CDK4	CTNNA1
CDK6	CTNNB1
CDKN1A	CUL3
CDKN1B	CUL4A
CDKN2A	CXCR4
CDKN2C	CYP17A1
CDX2	DAXX
CEBPA	DDR1
CHD1	DDR2
CHD4	DIS3
CHEK1	DNMT3A
CHEK2	DOT1L
CIC	EED
CNOT3	EGFR
COL2A1	EP300
CREBBP	EPHA3
CRLF2	EPHB1
CSF1	EPHB4
CSF1R	ERBB2
CSF3R	ERBB3

CSMD1	ERBB4
CTCF	ERCC4
CTNNA1	ERG
CTNNB1	ERRFI1
CUL3	ESR1
CUX1	EZH2
CXCR4	FAM46C
CYLD	FANCA
DAXX	FANCC
DDR2	FANCG
DDX3X	FANCL
DEPDC5	FAS
DGCR8	FBXW7
DIAPH2	FGF10
DICER1	FGF12
DLG2	FGF14
DMD	FGF19
DNM2	FGF23
DNMT3A	FGF3
DOCK8	FGF4
DPYD	FGF6
DROSHA	FGFR1
EEF1A1	FGFR2
EGFR	FGFR3
EIF1AX	FGFR4
ELF3	FH
EML4	FLCN
EP300	FLT1
EPAS1	FLT3
EPCAM	FOXL2
EPHA2	FUBP1
ERBB2	GABRA6
ERBB3	GATA3

ERBB4	GATA4
ERCC2	GATA6
ERF	GNA11
ERG	GNA13
ESR1	GNAQ
ETNK1	GNAS
ETS2	GRM3
EYS	GSK3B
EZH2	H3F3A
FANCL	HDAC1
FAT1	HGF
FAT4	HNF1A
FBXL17	HRAS
FBXO11	HSD3B1
FBXW7	ID3
FGF3	IDH1
FGFR1	IDH2
FGFR2	IGF1R
FGFR3	IKBKE
FGFR4	IKZF1
FH	INPP4B
FHIT	IRF2
FLCN	IRF4
FLT1	IRS2
FLT3	JAK1
FLT4	JAK2
FNTB	JAK3
FOSL2	JUN
FOXA1	KDM5A
FOXA2	KDM5C
FOXL2	KDM6A
FOXO3	KDR
FOXP1	KEAP1

FOXQ1	KEL
FUBP1	KIT
G6PD	KLHL6
GATA1	KMT2A (MLL)
GATA2	KMT2D (MLL2)
GATA3	KRAS
GATA6	LTK
GMDS	LYN
GNA11	MAF
GNAQ	MAP2K1 (MEK1)
GNAS	MAP2K2 (MEK2)
GPHN	MAP2K4
GPS2	MAP3K1
GRIN2A	MAP3K13
GSK3B	MAPK1
GSTP1	MCL1
GTF2I	MDM2
H3F3A	MDM4
H3F3B	MED12
HDHD1	MEF2B
HIF1A	MEN1
HIST1H1C	MERTK
HIST1H3B	MET
HIST2H3D	MITF
HLA-A	MKNK1
HLA-B	MLH1
HLA-C	MPL
HNF1A	MRE11A
HNRNPA2B1	MSH2
HOXC13	MSH3
HRAS	MSH6
IDH1	MST1R
IDH2	MTAP

IGF1R	MTOR
IGLL5	MUTYH
IKBKB	MYC
IKZF1	MYCL (MYCL1)
IL1RAPL1	MYCN
IL6ST	MYD88
IL7R	NBN
IMMP2L	NF1
IRF2	NF2
IRS2	NFE2L2
ITGA9	NFKBIA
ITGAV	NKX2-1
JAK1	NOTCH1
JAK2	NOTCH2
JAK3	NOTCH3
JMJD4	NPM1
KANSL1	NRAS
KCNJ5	NT5C2
KDM5C	NTRK1
KDM6A	NTRK2
KDR	NTRK3
KEAP1	P2RY8
KIT	PALB2
KLF4	PARK2
KLF5	PARP1
KMT2A	PARP2
KMT2B	PARP3
KMT2C	PAX5
KMT2D	PBRM1
KRAS	PDCD1 (PD-1)
KRT5	PDCD1LG2 (PD-L2)
KRTAP5-5	PDGFRA
LATS2	PDGFRB

LINC00290	PDK1
LINC01001	PIK3C2B
LMBRD2	PIK3C2G
LRP1B	PIK3CA
LRRN3	PIK3CB
LSAMP	PIK3R1
LZTR1	PIM1
MACROD2	PMS2
MAP2K1	POLD1
MAP2K2	POLE
MAP2K4	PPARG
MAP2K7	PPP2R1A
MAP3K1	PPP2R2A
MAP3K13	PRDM1
MAX	PRKAR1A
MCL1	PRKCI
MDM2	PTCH1
MDM4	PTEN
MECOM	PTPN11
MED12	PTPRO
MEGF6	QKI
MEN1	RAC1
MET	RAD21
MGA	RAD51
MGMT	RAD51B
MIPOL1	RAD51C
MITF	RAD51D
MLH1	RAD52
MLK4	RAD54L
MLLT4	RAF1
MPL	RARA
MSH2	RB1
MSH6	RBM10

MST1R	REL
MTHFR	RET
MTOR	RICTOR
MUTYH	RNF43
MYC	ROS1
MYCL	RPTOR
MYCN	SDHA
MYD88	SDHB
MYOD1	SDHC
NAALADL2	SDHD
NCOA2	SETD2
NCOR1	SF3B1
NEGR1	SGK1
NF1	SMAD2
NF2	SMAD4
NFE2L2	SMARCA4
NFKBIE	SMARCB1
NIPBL	SMO
NOTCH1	SNCAIP
NOTCH2	SOCS1
NPM1	SOX2
NRAS	SOX9
NRG1	SPEN
NSD1	SPOP
NT5C2	SRC
NTHL1	STAG2
NTRK1	STAT3
NTRK2	STK11
NTRK3	SUFU
OR11H1	SYK
OR4F21	TBX3
OR4N2	TEK
PABPC1	TERC

PALB2
PARD3
PARD3B
PARD6G
PARK2
PAX5
PBRM1
PDE4D
PDGFRA
PDGFRB
PDYN
PHF6
PHOX2B
PIK3CA
PIK3R1
PIK3R4
PIM1
PLCG1
PLXNB2
PML
PMS2
POLD1
POLE
POT1
PPM1D
PPP1R15A
PPP2R1A
PPP2R2A
PPP2R3B
PPP6C
PRDM1
PREX2
PRKACA
TERT
TET2
TGFBR2
TIPARP
TNFAIP3
TNFRSF14
TP53
TSC1
TSC2
TYRO3
U2AF1
VEGFA
VHL
WHSC1
WHSC1L1
WT1
XPO1
XRCC2
ZNF217
ZNF703

PRKAR1A
PSIP1
PTCH1
PTEN
PTK6
PTPN11
PTPN13
PTPRB
PTPRD
PTPRN2
RAC1
RACGAP1
RAD21
RAD50
RAD51B
RAD51C
RAD51D
RAD54L
RAF1
RARG
RASA1
RB1
RBM10
RET
RHOA
RHOB
RNF111
RNF43
ROS1
RPL10
RPL22
RPL5
RPS6KA3

RSF1
RUNX1
RXRA
RYR1
SAV1
SDHA
SDHAF2
SDHB
SDHC
SDHD
SEMG2
SETBP1
SETD2
SF3B1
SGCZ
SH2B3
SIX1
SIX2
SLCO1B1
SMAD2
SMAD3
SMAD4
SMARCA1
SMARCA4
SMARCB1
SMARCD1
SMC3
SMO
SMTNL2
SOCS1
SOX4
SOX9
SPATA31A7

SPEN
SPOP
SPRED1
SPTAN1
SRC
SRSF2
STAG2
STAT3
STAT5B
STK11
STS
SUFU
TBL1XR1
TBX3
TCF12
TCF7L2
TENM1
TERT
TET2
TG
TGFBR1
TGFBR2
TGIF1
TMEM30A
TMPRSS2
TNFAIP3
TNFRSF14
TOP1
TOP2A
TP53
TP63
TPMT
TPTE

TRAF7
TRIM51
TSC1
TSC2
TSHR
TYMS
U2AF1
UBR5
UGT1A1
USP22
USP28
USP32
USP8
VEGFA
VHL
WEE1
WT1
WWC3
WWOX
XPO1
XRCC1
ZBTB10
ZBTB20
ZBTB7B
ZFHX3
ZFP36L1
ZFP36L2
ZFX
ZIM3
ZMIZ1
ZMYM3
ZNF217
ZNF521

ZNF703
ZNF750
ZNRF3
ZRSR2
